# Effects of citrate-stabilized gold and silver nanoparticles on some safety parameters of *Porphyridium cruentum* biomass

**DOI:** 10.3389/fbioe.2023.1224945

**Published:** 2023-08-07

**Authors:** Ludmila Rudi, Liliana Cepoi, Tatiana Chiriac, Vera Miscu, Ana Valuta, Svetlana Djur

**Affiliations:** Phycobiotechnology Laboratory, Institute of Microbiology and Biotechnology of Technical University of Moldova, Chisinau, Moldova

**Keywords:** gold, silver, nanoparticles, *Porphyridium cruentum*, biomass, lipids, malondialdehyde, antioxidant activity

## Abstract

**Introduction:** Our research raises the question of how realistic and safe it is to use gold and silver nanoparticles in biotechnologies to grow microalgae, which will later be used to obtain valuable products. To this purpose, it was necessary to assess the influence of 10 and 20 nm Au and Ag nanoparticles stabilized in citrate on the growth of microalga *Porphyridium cruentum* in a closed cultivation system, as well as some safety parameters of biomass quality obtained under experimental conditions.

**Methods:** Two types of experiments were conducted with the addition of nanoparticles on the first day and the fifth day of the cultivation cycle. Changes in productivity, lipid content, malondialdehyde (MDA), as well as antioxidant activity of microalgae biomass have been monitored in dynamics during the life cycle in a closed culture system.

**Results:** The impact of nanoparticles on the growth curve of microalgae culture was marked by delaying the onset of the exponential growth phase. A significant increase in the content of lipids and MDA in biomass was noted. Excessive accumulation of lipid oxidation products within the first 24 h of cultivation resulted in altered antioxidant activity of red algae extracts.

**Discussion:** Citrate-stabilized gold and silver nanoparticles proved to be a stress factor for red microalga *Porphyridium cruentum*, causing significant changes in both biotechnological and biomass safety parameters. Addition of Au and Ag nanoparticles during the exponential growth phase of porphyridium culture led to an enhanced lipid accumulation and reduced MDA values in biomass.

## 1 Introduction

Gold and silver nanoparticles are among the most widely studied and applied in practice nanomaterials, being used in optoelectronics, catalysis, environmental protection (as remediation agents or as antimicrobials), food industry (food life extension, food packaging, and releasing of preservatives). These nanomaterials are used in the production of biosensors, targeted drug delivery systems, as well as in diagnostic imaging and cancer therapy (Majdalawieh et al., 2014; Patra et al., 2015; Ismail et al., 2017; Chugh et al., 2018).

The numerous areas in which gold and silver nanoparticles are applied and their increasing availability due to the development of engineering and bioengineering methods of synthesis lead to inevitable environmental pollution with these materials, which raises the question of their toxicity in relation to aquatic organisms, including microalgae (Moreno-Garrido et al., 2015).

Due to their rich and varied content of biologically active compounds, microalgae are considered valuable biotechnological objects. Some species are grown under industrial conditions to obtain nutraceuticals containing proteins, pigments, antioxidants, lipids, and carbohydrates. Nanoparticles display specific characteristics and absorption behavior in the aquatic environment, which can affect the growth and biosynthetic activity of microalgae both in their natural habitat and in bioreactors. For example, under controlled conditions, gold nanoparticles up to 10 nm in size can lead to an almost 2-fold increase in the carotenoid content in green algae biomass (Li et al., 2020). However, depending on the dose of gold nanoparticles to which the microalgae culture was exposed, as well as the surface properties of nanoparticles, the efficiency of photosystem II can either increase or decrease (Kula-Maximenko et al., 2022).

Marine microalga *Porphyridium cruentum* has raised particular interest due to its valuable biomass composition, which includes the following components per dry weight of algae: proteins (30%–35%), sulfated polysaccharides (20%–22%), phycobiliproteins (20%–25%), lipids (6%–8%) and numerous other compounds, present in small quantities (Rebolloso Fuentes, 2000). Among bioactive compounds with high commercial value are sulfated polysaccharides, phycobiliproteins and polyunsaturated fatty acids. Sulfated polysaccharides derived from *Porphyridium cruentum* biomass exhibit pronounced immunomodulatory and cytotoxic effects and have been proposed as natural and safe ingredients for nutraceuticals (Casas-Arrojo et al., 2021). Lipids produced by this red microalga are mostly polyunsaturated fatty acids, in particular arachidonic acid (C20:4, n-6) and eicosapentaenoic acid (C20:5, n-3), which can be exploited in various medical applications. Porphyridium biomass is also a precious source of natural antioxidants either for direct consumption or for use in food processing (Rodriguez-Garcia and Guil-Guerrero, 2008). This unicellular red alga has been used by several groups of researchers, including the authors of this article, within nanobiotechnological-based research where *Porphyridium cruentum* turned out to be an effective matrix for the synthesis of silver nanoparticles (Bunghez et al., 2010; Cepoi et al., 2021). In addition, some aspects of the interaction of *P. cruentum* culture with silver nanoparticles stabilized in polyethylene glycol and citrate were studied, and the effect of stimulating the accumulation of proteins and carbohydrates in biomass was revealed (Cepoi et al., 2021).

The involved pathway of nanoparticles affecting metabolic processes in microalgae is oxidative stress, and its setting can change the level of synthesis and accumulation of certain components of interest in phycological biomass. However, along with valuable biomass components, induced oxidative stress leads to excessive accumulation of oxidation products of macromolecules, which can compromise the quality of microalgae biomass. In the case of *P. cruentum*, lipids are the first components subjected to stress, and malondialdehyde (MDA) is one of the end products of polyunsaturated fatty acids peroxidation in algal cells (Chen et al., 2008). The degree of oxidative stress caused by nanoparticles depends on the specific characteristics of gold and silver nanoparticles, especially the compounds used to stabilize them. Moreover, stabilization of noble metal nanoparticles is crucial to maintaining the desired plasmonic behavior, hence various methods have been developed to stabilize nanomaterials, including citrate as a stabilizing layer (Kang et al., 2019). Thus, depending on the properties of nanoparticles and the type of cells that interact with them, numerous beneficial and negative effects can be observed.

The aim of our research was to assess the influence of 10 and 20 nm Au and Ag nanoparticles stabilized in citrate on the growth of *Porphyridium cruentum* culture during the cultivation cycle in a closed system, as well as some safety parameters of the quality of microalgae biomass obtained under experimental conditions.

## 2 Materials and methods

### 2.1 Gold and silver nanoparticles

To implement the design of our experimental studies, we used citrate-stabilized Au and Ag nanoparticles with diameters of 10 and 20 nm (SIGMA-ALDRICH CHEMIE GmbH, Germany). Product No for AuNP 10 nm–741957, and Product No for AuNP 20 nm–741965. Specifications: OD 1 and PDI <0.2. Product No for AgNP 10 nm–730785, and Product No for AgNP 20 nm–730793. Specification: 10 nm and 20 nm particle size (TEM). The characteristics of the nanoparticles are accurate and did not require additional measurements, the error in the size of nanoparticles is ±0.2 nm.

### 2.2 *Porphyridium cruentum* strain, nutrient medium, and cultivation conditions

The researches were carried out on *Porphyridium cruentum* CNMN-AR-01 strain (porphyridium), obtained from the National Collection of Nonpathogenic Microorganisms of the Institute of Microbiology and Biotechnology of Technical University of Moldova. Cultivation of *Porphyridium cruentum* CNMN-AR-01 was performed on mineral nutrient medium with the following composition: macroelements (SIGMA-ALDRICH CHEMIE GmbH, Germany): 16.04 g/L KCl; 12.52 g/L NaCl; 1.24 g/L KNO_3_; 2.5 g/L MgSO_4_·7H_2_O; 0.118 g/L CaCl_2_; 0.5 g/L K_2_HPO_4_·3H_2_O; 0.05 g/L KI; 0.05 g/L KBr. The microelements (SIGMA-ALDRICH CHEMIE GmbH, Germany) solution containing 2.86 mg/L H_3_BO_3_; 1.81 mg/L MnCl_2_·4H_2_O; 0.08 mg/L CuSO_4_·5H_2_O; 0.015 mg/L MoO_3,_ and the 0.5 mL FeEDTA solution. Microalgae cultivation was performed in 100 mL Erlenmeyer flasks (PYREX, Corning, New York, purchased from MERCK, Germany), with a working volume of 50 mL. The cultivation parameters regulating algal growth under laboratory conditions were strictly maintained: the inoculum size–0.5–0.55 g/L dry biomass; a temperature of 25–28°C, optimum pH in culture medium 6.8–7.2, and continuous illumination of 56 µM photons m^-2^ s^-1^. The cultivation cycle lasted 14 days.

### 2.3 Design of experiments

In the experiments involving the addition of gold and silver nanoparticles from the first day of the cultivation cycle of *Porphyridium cruentum*, Au and Ag nanoparticles, in the selected concentrations, were added to the mineral medium, followed by the inoculation of microalgae. In experimental samples, under the influence of nanoparticles during the exponential phase of porphyridium growth, Au and Ag nanoparticles at selected concentrations as stimulators were introduced into the nutrient medium on the 5th day of the cultivation cycle.

In samples monitoring changes in lipid and MDA content, as well as antioxidant activity, biomass was collected every 24 h.

Biomass collected from all experiments was demineralized [washed from excess salts with a solution of 2.0% ammonium acetate (SIGMA-ALDRICH CHEMIE GmbH, Germany)], standardized to a biomass concentration of 10 mg/mL per sample, and subjected to freezing/thawing procedures.

### 2.4 Determination of the biomass content

The biomass content of *P. cruentum* was determined spectrophotometrically by recording the absorption (Spectrophotometer T80 UV/VIS, PG Instruments, UK) of microalgal suspension at 545 nm and quantitative recalculation (g/L) was performed on the basis of the calibration curve.

### 2.5 Quantification of total lipids

The amount of total lipids was determined in the microalgal biomass spectrophotometrically based on the color reaction between the degradation products of lipids and the components of the phospho-vanillin reagent. To extract lipids from the microalgal biomass, 1.0 mL of chloroform (Merck KGaA (Supleco), Germany)/ethanol (Dita EstFarm SRL, Republic of Moldova) mixture (2v/1v) was added to 10 mg of biomass. The extraction was conducted by stirring (Heidolph Unimax 1010 shaker, Heidolph Instruments GmbH & Co. KG, Germany) at room temperature for 120 min. After the time elapsed, the lipid extract was separated from the other constituents, and 1.0 mL of 0.9% NaCl was added. This mixture was initially stirred and then centrifuged at 13520 g (Hettich centrifuge MIKRO 22R, Andreas Hettich GmbH & Co. KG, Germany). The supernatant was removed. Chloroform evaporated quite easily from the extracts. In the resulting precipitate was added 1.0 mL of concentrated sulfuric acid (Merck KGaA, Germany). For mixing, the samples were placed on a shaker and then transferred to water bath (Gesellschaft für Labortechnik mbH, Germany) at a temperature of 90°C for 20 min. The samples were then cooled under tap water. In dry glass tubes, 0.1 mL lipid extract hydrolysate and 3.0 mL phospho-vanillin reagent (1.2 mg vanillin (Merck KGaA (Supleco), Germany) in 1.0 mL of 68% phosphoric acid (Merck KGaA (Supleco), Germany) were mixed. The mixture of the reactants was kept in darkness at room temperature for 30 min. After incubation in the dark, the absorbance of the samples was recorded at the wavelength of 520 nm with respect to black. The lipid content (% biomass) was calculated on the basis of the calibration curve for pure oleic acid.

### 2.6 Determination of malondialdehyde content

To determine the content of malondialdehyde (MDA), 3.0 mL of 0.76% thiobarbituric acid (SIGMA-ALDRICH CHEMIE GmbH, Germany) in 20% trichloroacetic acid (SIGMA-ALDRICH CHEMIE GmbH, Germany) was added to 10 mg of biomass. The mixture of reactants was incubated on the water bath at 95°C for 40 min. Next, samples were cooled and centrifuged at 13520 g. The absorbance of samples was recorded at 532 nm and 600 nm. The mixture with no biological material was used as a blank sample. Quantitative analysis of malondialdehyde (nM/mL) in the samples was performed using the molar extinction coefficient of MDA-TBA product, which has a maximum absorption peak at 532 nm.

### 2.7 Evaluation of antioxidant activity

Antioxidant activity of freshly collected biomass was determined using the ABTS [2,2′-azinobis (3-ethylbenzothiazoline-6-sulfonic acid)] (SIGMA-ALDRICH CHEMIE GmbH, Germany) in ethanolic extracts derived from microalgal biomass. To prepare the alcoholic extracts, 1.0 mL of biomass with a concentration of 10 mg/mL was centrifuged at 13520 g for 10 min. The supernatant was removed, and 2.0 mL of 55% ethyl alcohol was added to the resulting biomass precipitate. The mixture was stirred at room temperature for 120 min and then centrifugated at 13520 g for 5 min. The obtained extracts were stored at +4°C. ABTS was generated by reacting equal volumes of 7 mM ABTS with 2.45 mM potassium persulfate (SIGMA-ALDRICH CHEMIE GmbH, Germany). The mixture was kept in the dark for 12–16 h, the time required for the formation of ABTS radical. Next, a working solution was prepared with an optical density of 0.700 ± 0.02 at 734 nm. The mixture of reactants consisted of 0.3 mL antioxidant extract and 2.7 mL ABTS. The samples were mixed and after 6 min their optical density was recorded at 734 nm. Antioxidant activity was expressed in % inhibition of ABTS.

### 2.8 Statistical analysis

The results were analyzed using Microsoft Excel, with the Student’s t-test and the correlation coefficient calculation. The threshold for statistical significance was *p* < 0.01. The investigations were performed in three independent experiments. For each parameter were performed three parallel measurements from each sample. Mean values along with standard deviation (S.D.) and p-values are presented in figures and tables.

## 3 Results

### 3.1 Amount of accumulated biomass

**FIGURE 1:**
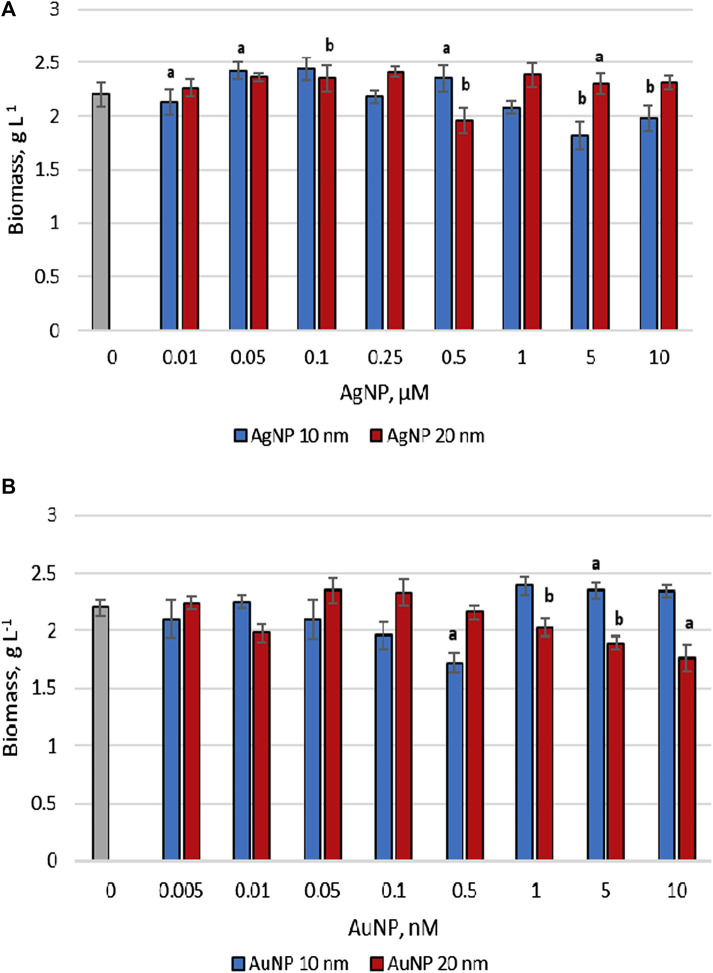
The biomass content accumulated by *Porphyridium cruentum* upon exposure to AgNP **(A)** and AuNP **(B)** (0–control), a -*p* < 0.01; b -*p* < 0.001.

The change in the amount of *P. cruentum* biomass accumulated at the end of the cultivation cycle in the experiments with the application of nanoparticles compared to control is shown in Figure 1. In the case of AgNPs, only those of 10 nm in size at concentrations of 5 and 10 μM reduced the amount of porphyridium biomass accumulated during the growth cycle. In other experiments, their effect was noted as favorable in terms of lipid synthesis or at least neutral (Figure 1A). Addition of 10 nm AuNPs in two of the tested concentrations, namely 0.1 and 0.5 nM, showed a 10.1%–20.2% (*p* < 0.01) reduction in microalgae biomass (Figure 1B). In the other studied cases, the effect of 10 nm gold nanoparticles stabilized in citrate on the amount of porphyridium biomass was neutral. Gold nanoparticles with a diameter of 20 nm at concentrations up to 1 nM did not produce significant changes on monitored parameter, while concentrations of 5 nM and 10 nM led to a decrease in the amount of biomass by 14.1 (*p* < 0.001) −20.2% (*p* < 0.01).

### 3.2 The amount of lipids and malondialdehyde at the end of the cultivation cycle

**FIGURE 2:**
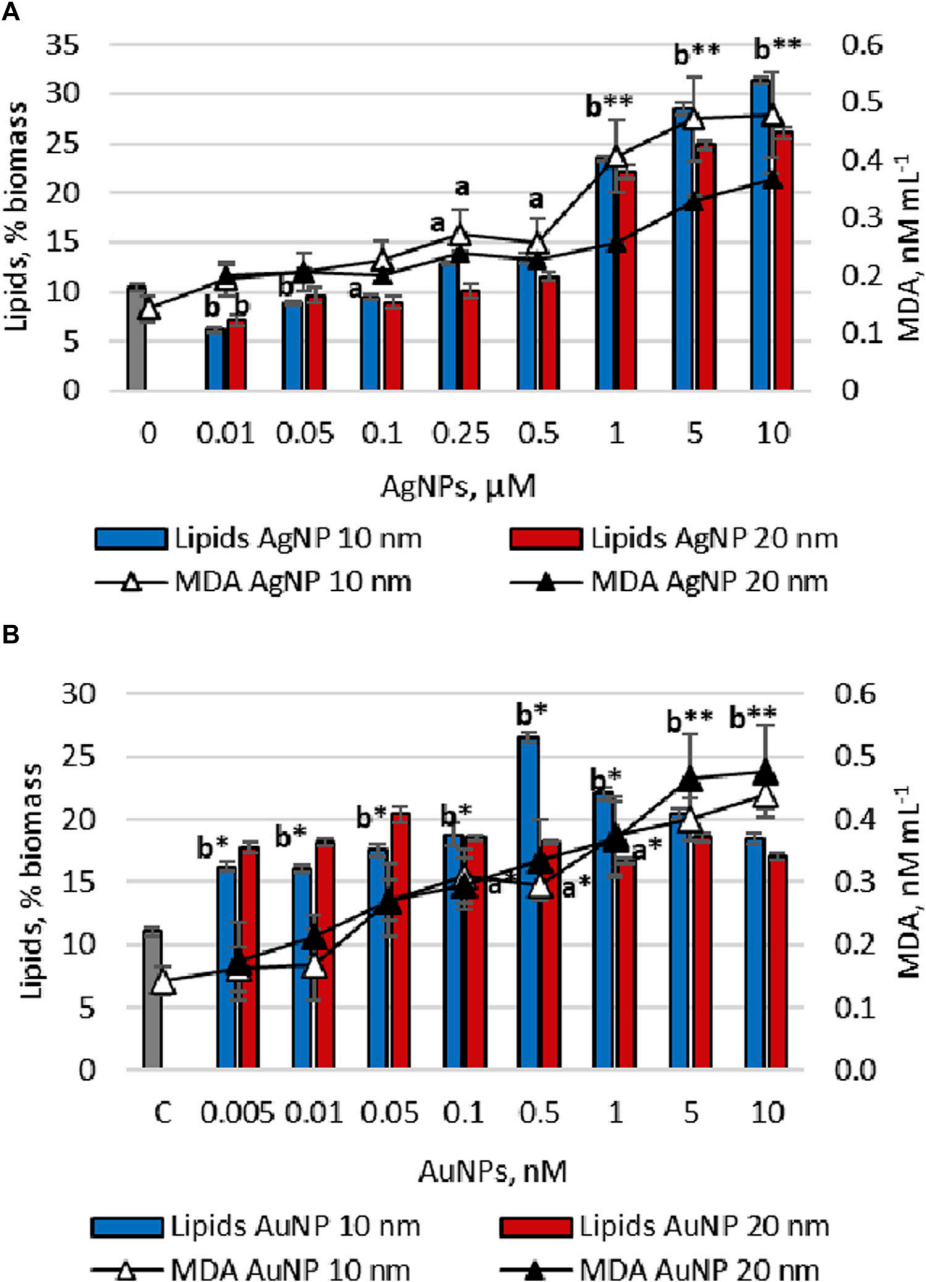
Total lipids and MDA content accumulated by *Porphyridium cruentum* upon exposure to AgNP **(A)** and AuNP **(B)** (0–control), a - *p* < 0.01; b -*p* < 0.001; a*-*p* < 0.01 common for MDA parameter; b*-p<0.001 common for lipids parameter; b**-*p* < 0.001 common for all parameters.

The effects of citrate-stabilized gold and silver nanoparticles on the lipid and malondialdehyde content in *Porphyridium cruentum* biomass at the end of the cultivation cycle can be observed in Figure 2.

The exposure of *Porphyridium cruentum* culture to Ag nanoparticles of both sizes in the concentration range from 0.01 to 0.1 μM resulted in a decrease in the lipid content in biomass (by up to 41.0% compared to control) (Figure 2A). Further, as supplementation with silver nanoparticles increased in the cultivation medium, lipid accumulation in biomass was also detected. Thus, the addition of 10 nm AgNPs in concentrations of 0.25 and 0.5 μM stimulated lipid synthesis by 23.4%–27.7% (*p* < 0.01) compared to control. Concentrations of 1–10 μM nanoparticles induced an increase in lipid content in microalgal biomass by 2.23–2.99 times (*p* < 0.001). In the case of 20 nm AgNPs, the lipid content increased 2.10–2.48 times (*p* < 0.001).

AgNPs exerted a concentration-dependent effect on the content of MDA in *P. cruentum* biomass and showed an increasing tendency of values with increase in nanoparticle concentration. In the case of 10 nm AgNPs MDA content increased by 36.4%–236% (*p* < 0.001) compared to control, and for nanoparticles of 20 nm in size–by 40.8%–159% (*p* < 0.001).

In the case of AuNPs, a significant increase from 45.1 up to 139.9% compared to control in the amount of lipids in biomass was established, depending on the size and concentration of nanoparticles. When using 10 nm AuNPs, the maximum lipid content in biomass was 2.39 times (*p* < 0.001) higher at a concentration of 0.5 nM. Citrate-stabilized 20 nm gold nanoparticles had a weaker stimulatory effect on lipid production in algae culture than 10 nm nanoparticles (Figure 2B). It was also found that the levels of malondialdehyde were high with increasing concentration of nanoparticles in the nutrient medium. For 10 nm AuNPs, an increase in the amount of MDA in biomass by 13.6 (*p* < 0.01)-208.9% (*p* < 0.001) compared to control was determined, while for those of 20 nm in size–by 22.6 (*p* < 0.001)–236.1% (*p* < 0.001).

### 3.3 Dynamics of porphyridium biomass accumulation during a cultivation cycle

Concentrations were selected for each type of nanoparticles and the highest lipid content in microalgae biomass was determined. Thus, concentration of 10 μM was revealed for AgNPs of 10 nm and 20 nm in size, while for 10 nm AuNPs–0.5 μM, and for 20 nm AuNPs–0.05 μM.

**FIGURE 3:**
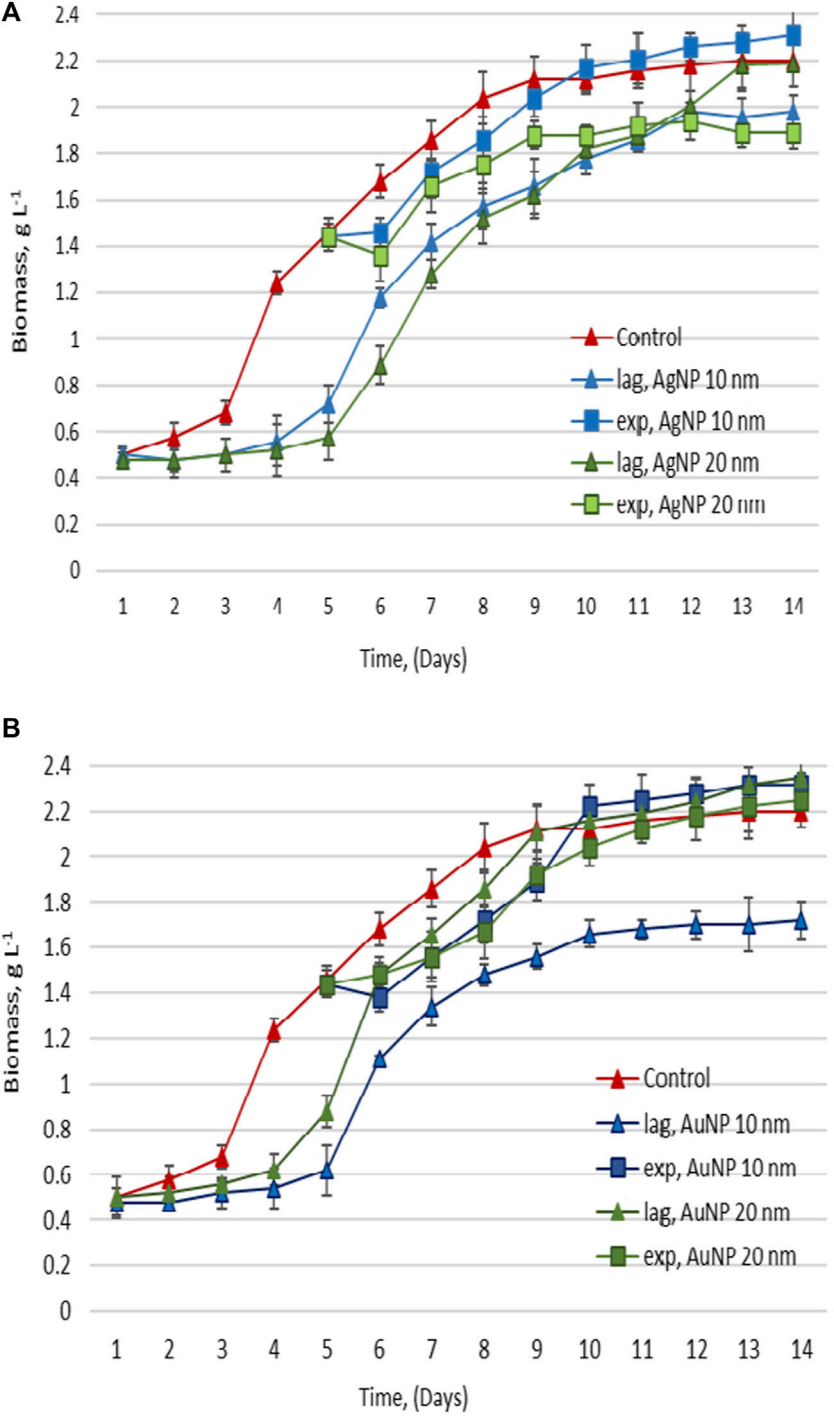
Biomass accumulated, upon the exposure of *Porphyridium cruentum* during the lag phase and exponential growth phase to 10 µM AgNP 10 nm and 20 nm **(A)**, and 0.5 nM AuNP 10 nm and 0.05 nM AuNP 20 nm **(B)**.

Nanoparticles were added to the culture medium on the first day of the cultivation cycle (lag phase of culture growth), and in the other experimental series–on the 5th day, which corresponded to the exponential growth phase. At equal 24-h intervals, samples of *Porphyridium cruentum* culture were taken to determine the amount of biomass, total lipid content and the values of malondialdehyde accumulated in biomass. The results with reference to the amount of produced biomass are shown in Figure 3.

In the case of adding AgNPs to the nutrient medium on the first day of cultivation, lag phase duration increased significantly (Figure 3A). The beginning of the exponential growth phase occurred on the 5th day and was clearly manifested on the 6th day of cultivation (2 days later than in control). In the case of 20 nm AgNPs, biomass amount in the experimental sample was equal to the one in control on the 13th day of cultivation, while in the case of 10 nm AgNPs, it remained significantly lower. When silver nanoparticles of both sizes were added on day 5 of cultivation, delays in the growth phases were avoided, with only a slight decrease in the amount of biomass during the exponential growth phase compared to control (Figure 3B).

If gold nanoparticles of both sizes were added to *P. cruentum* nutrient medium on the first day of vital cycle, lag phase duration also increased by 72 h. In the case of 20 nm AuNPs, the amount of biomass reached the control level on the 9th day of cultivation, and in the case of 10 nm AuNPs, algae biomass was significantly lower than in control sample (Figure 3A). The introduction of AuNPs on the 5th day of cultivation made it possible to avoid delays in the growth phases, and the decrease in the amount of biomass produced on subsequent days was exceeded at the end of the growth cycle (Figure 3B).

### 3.4 Dynamics of lipid and MDA accumulation in *Porphyridium cruentum* biomass during a cultivation cycle

**FIGURE 4:**
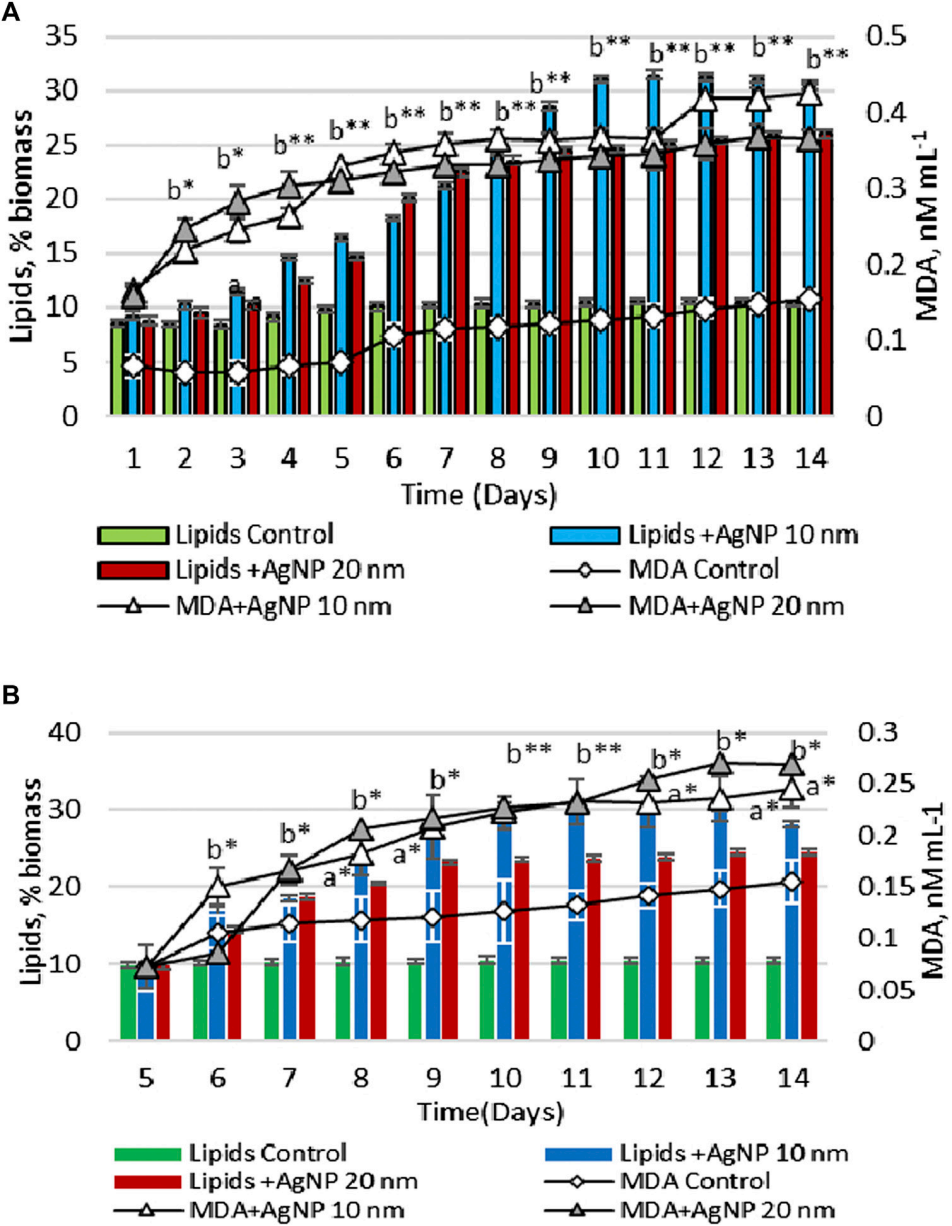
Total lipid and MDA contents in the biomass, upon the exposure of *Porphyridium cruentum* during the lag phase **(A)** and exponential growth phase **(B)** to 10 µM AgNP 10 nm and 10 µM AgNP 20 nm; a -*p* < 0.01; b -*p* < 0.001; a*-*p* < 0.01 common for two parameters; b*-*p* < 0.001 common for two parameters; b**-*p* < 0.001 common for all parameters.

Figure 4 represents the changes in the content of lipids and MDA in biomass during the growth cycle in experiments with the application of nanoparticles in the lag phase and the exponential growth phase. Table 1 shows the lipids and MDA amount calculated in biomass under the application of Au and Ag nanoparticles at different growth phases of cultivation.

**TABLE 1:** Lipids and MDA content in *Porphyridium cruentum* under the application of Ag and Au nanoparticles at different periods of the cultivation cycle (1st day–lag phase; 5th day–exponential growth phase).

Days	AgNPs, 10 nm		AgNPs, 20 nm		AuNPs, 10 nm		AuNPs, 20 nm	
	Lipids g/L	MDA, nM/L	Lipids, g/L	MDA, nM/L	Lipids, g/L	MDA, nM/L	Lipids, g/L	MDA, nM/L
Adding NPs on the first day of the cultivation cycle, ^a^ *p* < 0.01; ^b^ *p* < 0.001								
9	0.47 ± 0.040^a^	60.42 ± 5.69^b^	0.39 ± 0,028^b^	54.76 ± 5.16^a^	0.28 ± 0.014^a^	30.57 ± 2.43^b^	0.35 ± 0.026^b^	51.06 ± 5.86^a^
10	0.55 ± 0.028^a^	65.50 ± 4.53^b^	0.45 ± 0.028^b^	62.24 ± 4.74^a^	0.37 ± 0.019^a^	35.03 ± 2.18^a^	0.39 ± 0.020^b^	55.73 ± 4.66^a^
11	0.58 ± 0.023^b^	68.07 ± 4.24^b^	0.47 ± 0.023^b^	65.05 ± 6.21^a^	0.41 ± 0.016^a^	41.16 ± 1.53^b^	0.40 ± 0.020^b^	57.38 ± 4.20^b^
12	0.62 ± 0.012^b^	82.76 ± 2.02^b^	0.51 ± 0.022^b^	72.16 ± 6.38^a^	0.43 ± 0.019^a^	45.22 ± 2.34^b^	0.45 ± 0.026^b^	59.58 ± 6.06^b^
13	0.61 ± 0.030^b^	81.93 ± 6.28^b^	0.56 ± 0.036^b^	80.22 ± 7.75^a^	0.44 ± 0.038^b^	48.96 ± 4.79^a^	0.47 ± 0.026^b^	61.48 ± 4.87^b^
14	0.60 ± 0.030^b^	84.15 ± 5.15^b^	0.57 ± 0.032^b^	80.15 ± 6.29^a^	0.46 ± 0.027^a^	51.08 ± 4.40^a^	0.48 ± 0.026^b^	63.38 ± 6.98^a^
Adding NPs on the 5th day of the cultivation cycle, ^a^ *p* < 0.01; ^b^ *p* < 0.001								
9	0.57 ± 0.030^b^	42.43 ± 5.13^a^	0.44 ± 0.018^b^	40.60 ± 5.43^a^	0.49 ± 0.018^b^	31.37 ± 4.36 ^a^	0.45 ± 0.031^b^	23.42 ± 4.41
10	0.66 ± 0.039^b^	48.17 ± 6.78^a^	0.44 ± 0.017^b^	42.86 ± 4.10^a^	0.59 ± 0.020^b^	40.40 ± 3.76^a^	0.49 ± 0.024^b^	27.00 ± 3.52
11	0.68 ± 0.043^b^	51.49 ± 6.54^a^	0.45 ± 0.031^a^	44.54 ± 5.39^b^	0.64 ± 0.018^b^	44.10 ± 3.85^b^	0.53 ± 0.022^b^	54.00 ± 3.22
12	0.69 ± 0.027^b^	52.43 ± 8.39^a^	0.46 ± 0.026^b^	49.27 ± 6.49^a^	0.67 ± 0.029^b^	45.83 ± 5.03^b^	0.57 ± 0.036^b^	31.37 ± 5.74
13	0.69 ± 0.026^b^	53.80 ± 6.67^b^	0.46 ± 0.021^b^	51.22 ± 4.65^b^	0.69 ± 0.036^b^	47.56 ± 5.73^a^	0.59 ± 0.034^b^	35.97 ± 5.52^a^
14	0.65 ± 0.042^b^	56.59 ± 8.02^b^	0.46 ± 0.022^b^	51.03 ± 4.53^b^	0.70 ± 0.034^b^	46.63 ± 5.95^a^	0.60 ± 0.038^b^	39.74 ± 5.32^a^

Lipid content in the control sample was constant throughout the entire growth cycle and varied within 8.44%–10.56% of dry biomass. The content of MDA was maintained at a stable level during the first 5 days, after which on the 6th day it increased by 60.6% compared to the first day and remained at a higher level until the end of the cultivation cycle.

When AgNPs of both sizes were introduced on the first day, a significant increase in both the amount of lipids and the level of MDA in biomass was found during the vital cycle. On the second day, the amount of lipids and MDA in the biomass resulting from the experiment with nanoparticles was higher than in control sample by 21.5%–197.7% (*p* < 0.001) for lipids and 274.2%–355.5% (*p* < 0.001) for MDA. This difference has been increasing with the advancement of culture in age (Figure 4A).

In the case of adding silver nanoparticles on the 5th day of cultivation, an increase in the content of lipids and MDA was also detected. On day 14, lipid content in algae biomass grown on nutrient medium with 10 nm AgNPs was 2.69 times (*p* < 0.001) higher compared to control. The amount of lipids in microalgae culture was 0.60 g/L (Table 1). However, in this case, the increase of MDA level was less pronounced. On the 6th day of cultivation, MDA level was 42.3% (*p* < 0.001) lower than on the same day, but with the addition of nanoparticles on the first day (Figure 4B). At the end of the cultivation cycle, MDA content was 56.59 nM/L compared to 84.15 nM/L. A similar response pattern was observed for 20 nm AgNPs. The measurement of malondialdehyde content in microalgae culture with the addition of 20 nm AgNPs on the 5th day of cultivation was 51.03 nM/L compared to 80.15 nM/L MDA produced in the case of using nanoparticles on the first day of cultivation. The amount of lipids in microalgae culture was 0.46 g/L (Table 1).

**FIGURE 5:**
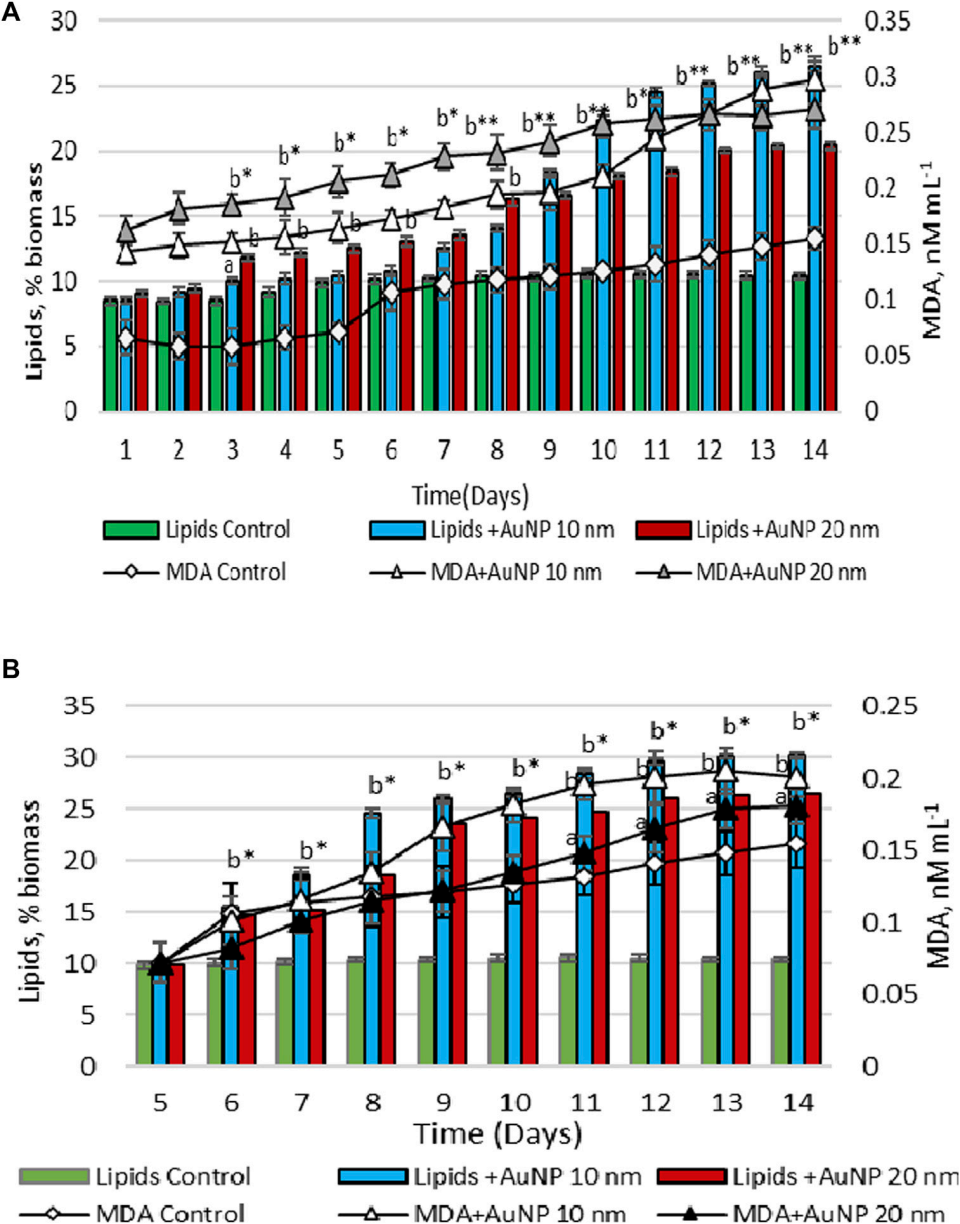
Total lipid and MDA contents in the biomass, upon the exposure of *Porphyridium cruentum* during the lag phase **(A)** and exponential growth phase **(B)** to 0.5 nM AuNP 10 nm and 0.05 nM AuNP 20 nm; a -*p* < 0.01; b -*p* < 0.001; b*-*p* < 0.001 common for two parameters.

The dynamics of changes in the content of lipids and MDA in *Porphyridium cruentum* biomass during the growth cycle in the presence of gold nanoparticles can be seen in Figure 5.

In porphyridium biomass grown on culture medium with the addition of 10 nm AuNPs on the first day, on the 14th day the lipid content was 2.54 times (*p* < 0.001) higher than the control value and amounted to 0.46 g/L (Figure 5A; Table 1). The content of MDA was higher than in control sample throughout the entire cycle of cultivation. AuNPs of 20 nm in size enhanced lipid content by 1.96 times (*p* < 0.001) compared to control (Figure 5A). The amount of lipids in microalgae culture at the end of cultivation was 0.48 g/L. The MDA content during lag phase and the exponential growth phase was higher towards the results obtained in the experiment with 10 nm AuNPs, but at the final harvesting of microalgae culture, the level of this parameter in biomass was the same. The content of MDA in microalgal biomass was 51.08 nM/L for 10 nm AuNPs and 63.38 nM/L for 20 nm AuNPs (Table 1).

Adding Au nanoparticles on the 5th day of the cultivation cycle led to an increase in the lipid content of the biomass compared to their application on the first day and exceeded the control level by 2.9 times (*p* < 0.001) for 10 nm AuNPs and 2.54 times (*p* < 0.001) for 20 nm AuNPs (Figure 5B). Lipid content amounted to 0.70 g/L and 0.60 g/L, respectively (Table 1). On the contrary, MDA content in porphyridium biomass was about 30% lower compared to the values determined in the experiment of using AuNPs on the first day of the cultivation cycle. The MDA content in *P. cruentum* biomass was 46.63 nM/L and 39.74 nM/L, respectively (Table 1).

### 3.5 Dynamics of antioxidant activity of hydro-ethanolic extract derived from porphyridium biomass exposed to the action of Au and Ag nanoparticles

**FIGURE 6:**
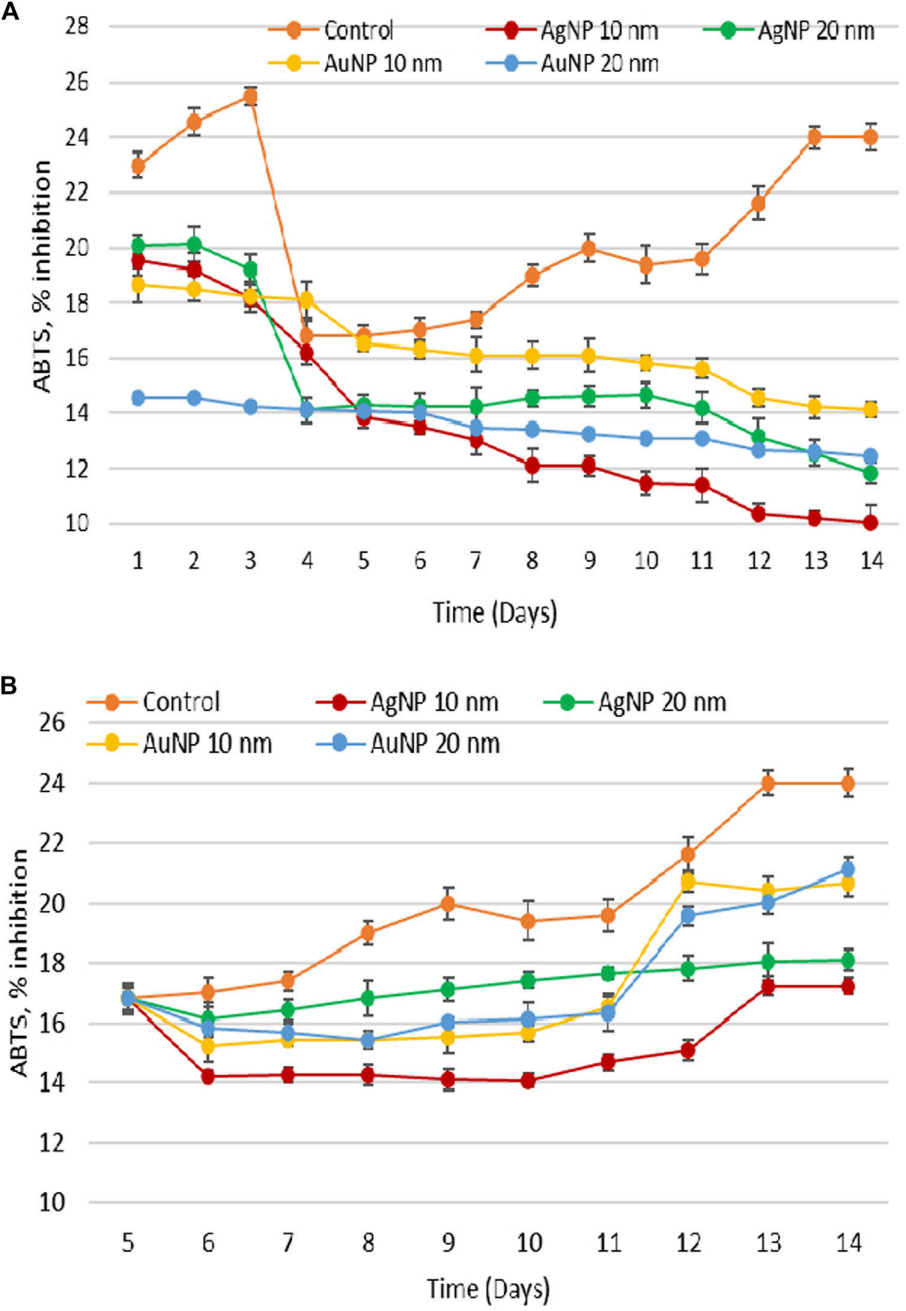
Change in antioxidant activity of ethanolic extract from *Porphyridium cruentum* exposed to Ag and Au nanoparticles (10 µM AgNP 10 nm; 10 µM AgNP 20 nm; 0.5 nM AuNP 10 nm; 0.05 nM AuNP 20 nm) during the lag phase **(A)** and exponential growth phase **(B)**.

Figure 6 shows the change in the antioxidant activity of hydro-ethanolic extract obtained from porphyridium biomass grown on culture medium with the addition of AgNPs and AuNPs on the first and fifth days of cultivation.

Under optimal laboratory conditions of cultivation, the highest ABTS values were characteristic to lag phase and related to the adaptation of inoculum to fresh nutrient medium. On the fourth day, the ABTS test values decreased significantly, followed by a steady increase until the end of cultivation cycle. The presence of gold and silver nanoparticles caused a significant decrease in antioxidant activity compared to control that persisted throughout the entire cultivation cycle, especially during the 12th to 14th days. The difference ranged from 20% to 30% in the exponential growth phase and from 40% to 60% in the stationary growth phase.

In the experiment with the addition of nanoparticles on day 5, the antioxidant activity of extracts derived from porphyridium biomass grown on culture medium with nanoparticles was also lower compared to control, but the difference was less obvious than in the experiment with the application of nanoparticles on the first day. At the same time, in this experiment, days 12–14 were characterized by an increase in antioxidant activity towards the values of the exponential growth phase. However, these values were lower compared to control.

## 4 Discussion

*Porphyridium cruentum* is a red microalgae of biotechnological importance, and lipids proved to be valuable components of its biomass. Synthesis and accumulation of large amounts of lipids can be enhanced by applying various biotechnological procedures. A prerequisite for this is biomass quality obtained under these conditions that must be safe for further use, especially for direct human use or as a raw material for the production of various preparations. In parallel with biomass production and lipid content therein, it is necessary to monitor changes in MDA levels, which will be used as a safety parameter within microalgae production schemes.

It is known that the amount of biomass accumulated during a growth cycle of microalgae is one of the main parameters for assessing the toxic effects of nanomaterials on these organisms. Depending on the type of nanomaterials, their concentration, and the organism under study, the effects can be either stimulatory or inhibitory. The size of nanoparticles plays a critical role in their interaction with the cell. In the case of microalgal cell walls, they are equipped with pores with a diameter ranging from 5 to 20 nm, which facilitates the penetration of nanoparticles of the same size (Navarro et al., 2008). Obtaining a stimulatory effect upon nanoparticle action without exhibiting toxicity is extremely important.

Observations regarding the effects of nanoparticles are diverse and contradictory when it comes to the relationship between concentration-dependent effects. There are cases where low concentrations of nanoparticles have shown positive effects, while high concentrations have exerted negative effects (Pádrová et al., 2015; Cepoi et al., 2020). In other cases, however, an inverse relationship has been revealed (Wang et al., 2021). In the context of nanoparticle effects on organisms, the notion of low and high concentrations refers more to the effects produced rather than the quantity. The concentrations of nanoparticles characterized as low have been found to stimulate lipid production (Vargas-Estrada et al., 2020). At the same time, the ranges of nanoparticle concentrations applied to microalgae are highly varied. For example, AgNPs were applied to *Chlamydomonas reinhardtii* culture in the concentration range of 10, 40, 75, 150, and 300 μg/L (Sendra et al., 2017), and in the concentration range of 0.1, 1.0, 10, 100, and 1,000 mg/L (Pedroso et al., 2013). All these studies aimed to determine the concentration limits at which a stimulatory effect on biosynthetic processes in microalgae can be established. Similarly, the concentration ranges of AuNPs and AgNPs presented in this research paper were analyzed.

Microalgal cells undergo various biological changes when facing adverse environmental stresses. Lipids are components of microalgae biomass that respond to various xenobiotic substances, including nanoparticles of different types. In this research, citrate-stabilized silver nanoparticles with 10 and 20 nm in diameter exhibited two types of effects on the amount of lipids in *P. cruentum* biomass: inhibitory effect characteristic at low nanoparticle concentrations, and stimulatory effect for high ones. However, within the limits of the applied concentrations of AgNPs, the correlation between their concentration in nutrient medium and lipid content in algae biomass collected on the 14th day of the life cycle was strong, the Pearson correlation coefficient calculated for 10 nm AgNPs was r = 0.856, and for 20 nm AgNPs–r = 0.819. A similar response was found under the growth conditions of mixotrophic *Chlorella* sp*.* UJ-3 in the presence of low concentrations of Fe_3_O_4_ nanoparticles in nutrient medium (Wang et al., 2021). In the case of AuNPs of both sizes, lipid content in biomass was significantly higher, but the effect was not concentration–dependent. Thus, within the limits of the used concentrations of AuNPs, it was found a weak negative correlation between the variables. The type of nanoparticles and their concentrations are determinant factors for promoting lipid synthesis in microalgae biomass. Thus, stimulatory effects of some nanoparticles on lipid accumulation have been revealed in other algal cultures treated with different types of nanoparticles: *Oedogonium* sp., *Cladophora* sp., *Ulothrix* sp., and *Spirogyra* sp. using AgNPs (Hasnain et al., 2023); *Scenedesmus obliquus* exposed to Fe_2_O_3_ and Fe_3_O_4_ nanoparticles (He et al., 2017; Wang et al., 2021); *Trachydiscus minutus*, *Desmodesmus subspicatus* and zerovalent iron (ZVI) nanoparticles (Pádrová et al., 2015); *Chlorella vulgaris* upon influence of TiO_2_ nanoparticles and MgNPs (Kang et al., 2014; Sibi et al., 2017).

Lipid production can be induced in microalgae in response to oxidative stress that appears in cells upon their contact with xenobiotics, but lipid oxidation process is also initiated. Malondialdehyde is one of the end products of the peroxidation of polyunsaturated fatty acids in cells and is commonly known as a biomarker of oxidative stress. In this research, there was revealed a strong positive correlation between malondialdehyde levels in *Porphyridium cruentum* biomass and nanoparticle concentrations in the nutrient medium. This was confirmed by correlation coefficients r = 0.823 for AgNPs 10 nm in size, and r = 0.899 for the experiment with 20 nm AgNPs; and in the case of using AuNPs of 10 and 20 nm, the Pearson values were r = 0.744 and r = 0.817, respectively. Increased MDA levels were determined in other algal cultures treated with different types of nanoparticles, for example in the study of the action of titanium dioxide (TiO_2_) nanoparticles on green alga *Chlorella pyrenoidosa* (Middepogu et al., 2018). Another scientific paper noted that AgNPs added to the growth media of microalgae *Chlorella vulgaris* and *Dunaliella tertiolecta* resulted in elevated values of malondialdehyde (Hazani et al., 2013).

The study of the parameters of interest in dynamics during the growth cycle of microalgae *Porphyridium cruentum* showed that gold and silver nanoparticles exerted visible effects on them. The most obvious was the change in the duration of culture growth phases with an increase in the length of *lag* phase and a reduction in the exponential growth phase when nanoparticles were introduced on the first day of cultivation. Except for 10 nm AuNPs, in the second part of the exponential growth phase of microalgae culture, the amount of biomass in the experiment was close to the control values, and accumulated lipid levels were very high. In parallel, a continuous increase was observed in the quantity of MDA and a gradual decrease in the antioxidant activity of porphyridium biomass. Thus, a state of pronounced oxidative stress was detected, which can be diminished by adding nanoparticles later in the growth cycle.

The introduction of nanoparticles on the 5th day of the growth cycle provided the optimal amount of biomass containing high quantities of lipids under conditions of significantly lower levels of MDA (compared to the experiment of adding NPs on the first day) and an adequate level of antioxidant activity of the biomass. The intensity of oxidative degradation processes of macromolecules, including lipids, was influenced by the ability of culture to produce antioxidants capable of scavenging free radicals, thereby breaking oxidation chains and preventing cellular damage. It is known that microalgae responded to different stressors by altering the antioxidant activity, which, depending on the stress intensity, can significantly increase or decrease (Dummermuth et al., 2003; Barone et al., 2021).

The correlation between MDA and ABTS values underlines the essential role of antioxidant components in controlling nanoparticle-induced oxidative stress. Thus, when adding nanoparticles during lag phase, we found a negative correlation, which indicated a low level of components with antioxidant properties. At the same time, when nanoparticles were introduced during the exponential growth phase of life cycle, the correlation between MDA and ABTS values was positive and reflected the involvement of antioxidant compounds in culture protection (Table 2).

**TABLE 2:** Pearson coefficient calculated for the relationship between MDA values and the antioxidant activity of *Porphyridium cruentum* biomass, depending on the age at which the culture comes into contact with the nanoparticles (lag phase and exponential growth phase) (Duration of the cultivation cycle 14 days).

Growth phase in which the NPs were added	Correlation coefficient value			
	AgNP 10 nm	AgNP 20 nm	AuNP 10 nm	AuNP 20 nm
Lag growth phase	−0.98424	−0.88383	−0.92451	−0.97555
Exponential growth phase	0.845999	0.941365	0.741151	0.874284

According to the data above, it can be concluded that within the described experimental conditions, the most favorable proceedings for obtaining *P. cruentum* biomass with high lipid content are those using 10 nm silver and gold nanoparticles, supplemented during the exponential growth phase. Biomass harvesting should be carried out on the 12th day of the cultivation cycle for estimating its safety in terms of reaching the balance between the main biotechnological parameter - content of lipids of 0.69 g/L for AgNPs and 0.67 g/L for AuNPs, and biomass safety parameter - MDA level of 52.43 nM/L for AgNPs and 45.83 nM/L for AuNPs (Table 1).

## 5 Conclusion

Citrate-stabilized gold and silver nanoparticles 10 and 20 nm in size were a stress factor for red microalgae *P. cruentum*, causing significant changes in both biotechnological and biomass safety parameters. They can be used as factors to stimulate lipid accumulation by microalgae. In the case of using biomass or lipids derived from porphyridium for humans, it is necessary to consider the intensity of lipid oxidation processes so that the end product does not contain increased amounts of oxidative degradation products. From a technological point of view, this balance can be achieved by identifying the type of material used as a stimulator, its concentration, and the phase of the growth cycle at which it is introduced into the biotechnological process.

## Data Availability

The original contributions presented in the study are included in the article/supplementary material, further inquiries can be directed to the corresponding author.
